# Supplementary material for: Dependency of Tamoxifen Sensitive and Resistant ER+ Breast Cancer Cells on Semaphorin 3C (SEMA3C) for Growth
Source: Cells. 2023 Jun 25;12(13):1715. doi: 10.3390/cells12131715 (PMC10341167; doi:10.3390/cells12131715)
Supplement: Supplementary file 1 [file cells-12-01715-s001.zip › cells-2410287-supplementary.pdf]

S1.

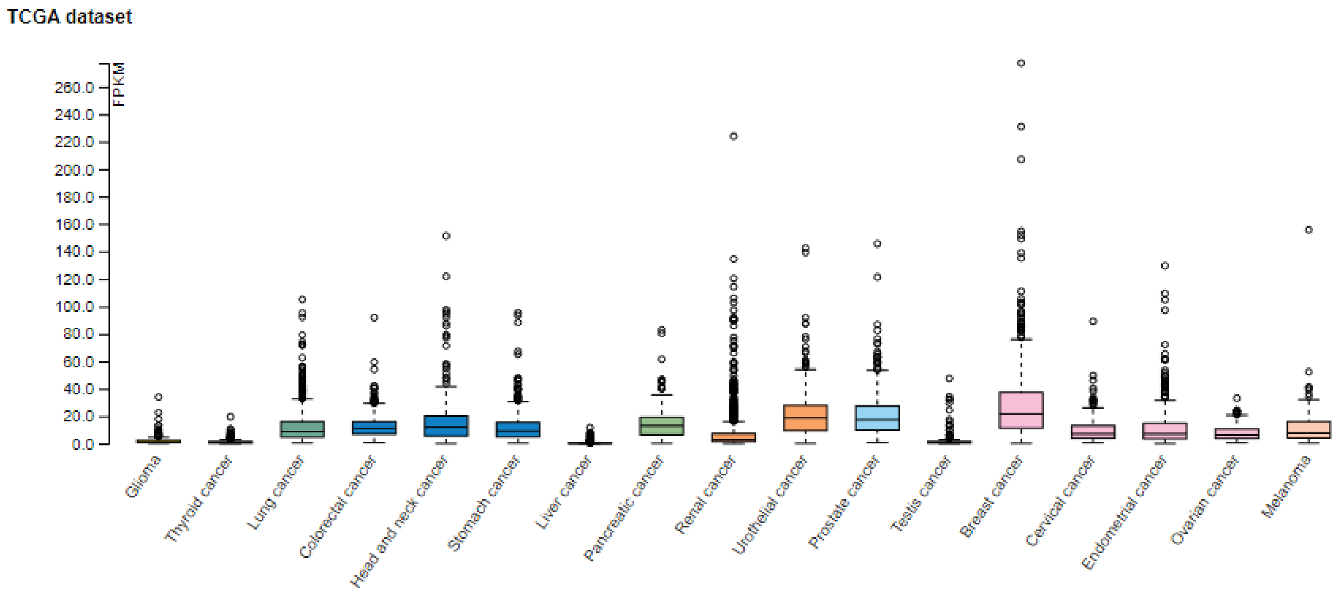

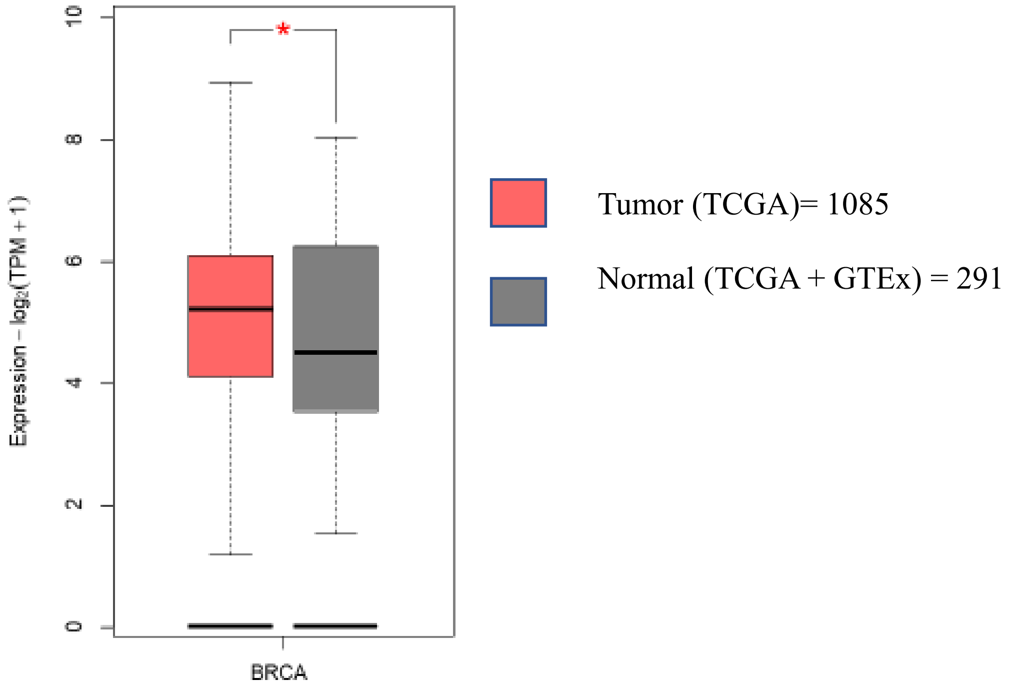

Figure S1. RNA expression of SEMA3C is higher in breast cancer across different tumors in TCGA dataset available on protein atlas (image derived from protein atlas) and comparison of SEMA3C mRNA expression between breast tumor (n=1085) and normal (n=291) samples from TCGA and GTEx dataset reveals higher mean expression of SEMA3C in tumor samples (Log_2FC threshold >0.5) using GEPIA2. *$p<0.05$

S2.

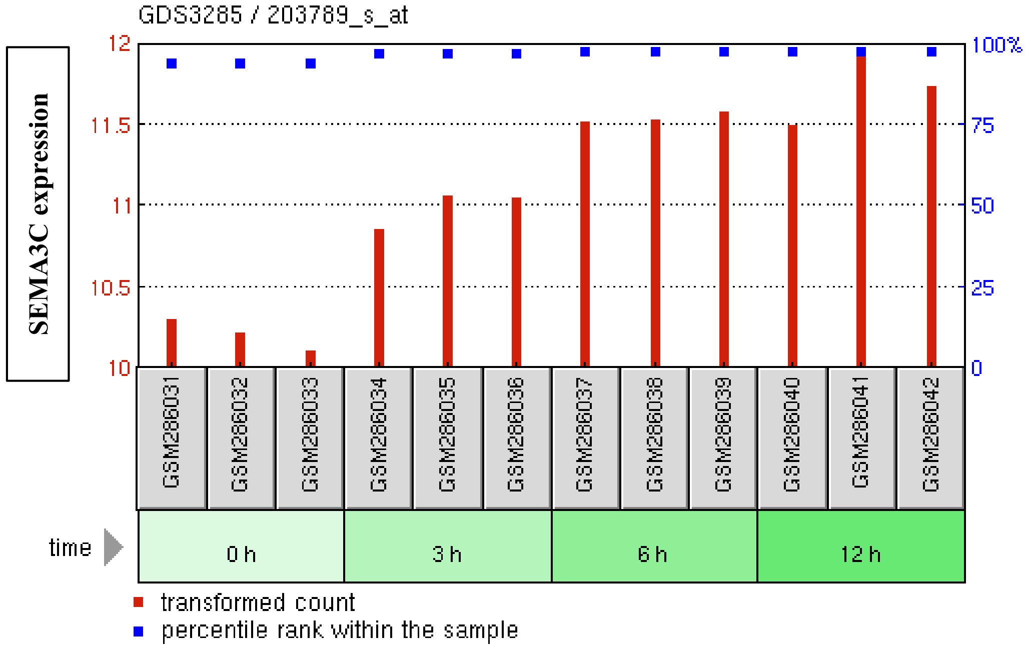

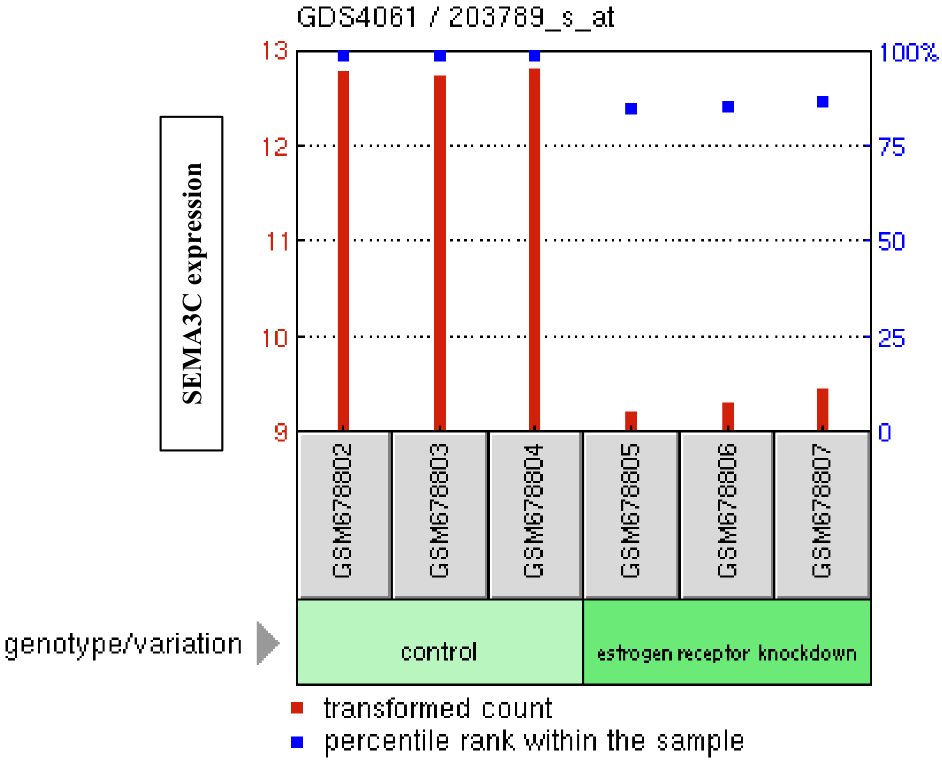

Figure S2. mRNA expression of SEMA3C increases in a time dependent manner under estradiol treatment (100nM) as analyzed in GSE11324 and significant decrease in SEMA3C expression during ESR1 knockdown as analyzed in GSE27473.

Densitometry Analysis

S3.

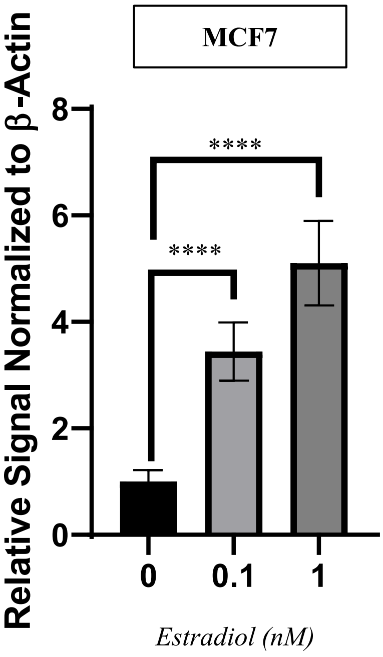

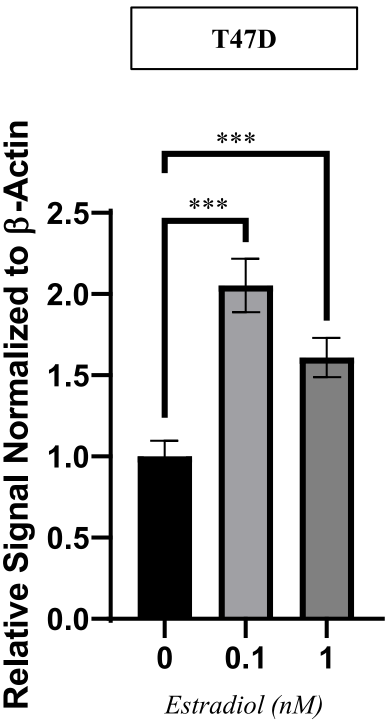

Figure S3. Quantification of SEMA3C protein levels in MCF7 and T47D cells after an overnight treatment with estradiol in a dose dependent manner. ***$p<0.001$;****$p<0.0001$

Table S1.

Regulatory Region	Positive Strand	Negative Strand
Distal Enhancer<br>chr7:80,940,030-80,944,498	• GTACAAGCTGACCCTGAGCC	• TGGATCAAAGAGACATT<br>• GCTCAGGGTCAGCTTGTAC
Promoter+ Intron 1<br>Chr7:80,919,983-80,922,983	• AGGGCCAGGATGGTCTG	• AAGCATGAGCACTGTGTCCTT<br>• GATCCAGACCATCCTGGCCC
Intron 2<br>chr7:80,876,356-80,877,911	• AGGGTCACGCCAAACTG	• CAGGTTACAAAGAGCTT

Table S1. ESR1 binding peaks region derived from ChIP-Seq studies on SEMA3C locus showcasing ERE motifs in all three regulatory regions on both positive and negative strand.

S4.

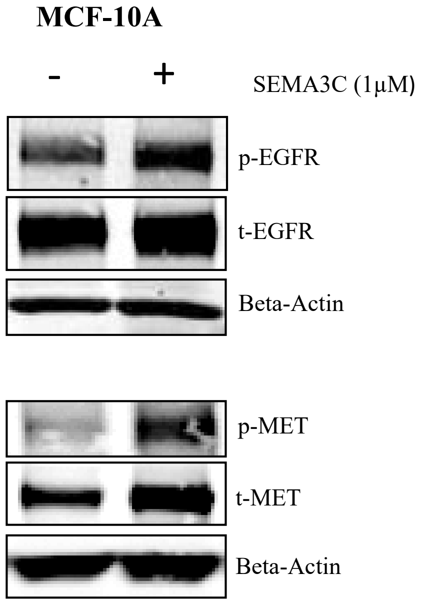

Figure S4. Exogenous treatment with recombinant SEMA3C (1uM) for 10 minutes on MCF10A leads to increase in phosphorylation of EGFR and MET.

S5.

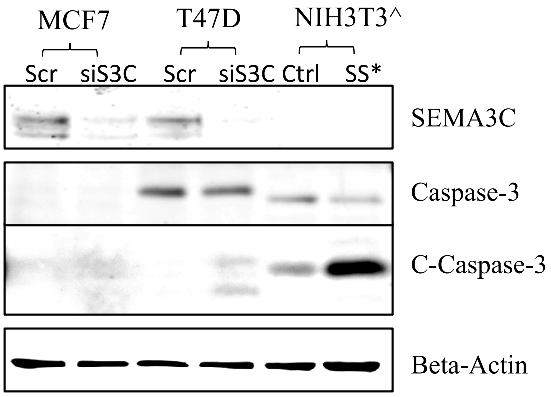

*Staurosporine

^ Positive Control for Caspase-3 detection

Figure S5. Confirmation of absence (MCF7) and presence (T47D) of caspase-3 dependent pro-apoptotic signal post SEMA3C siRNA treatment (25nM) benchmarked with positive control used for caspase-3 and cleaved caspase-3 detection (NIH3T3).

S6.

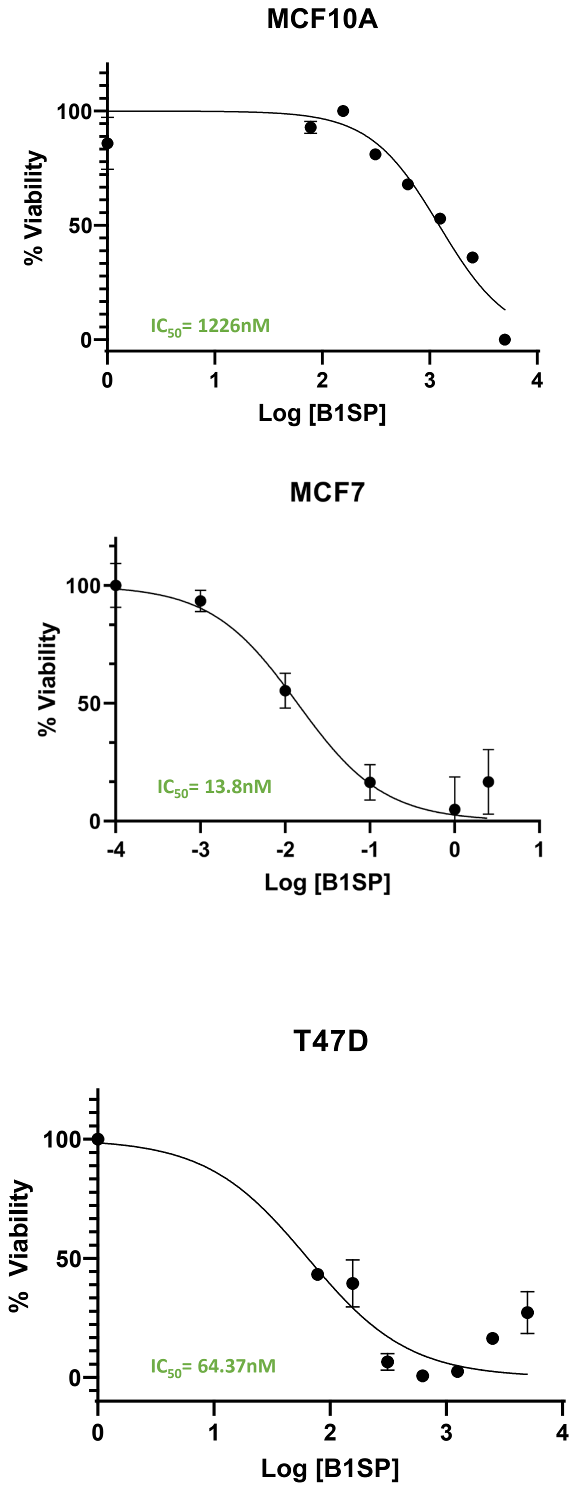

Figure S6. IC50 log curves of B1SP on normal mammary epithelial cells (MCF10A) and ER+ Breast Cancer cells (MCF7 and T47D). Based on IC50 values, B1SP has higher specificity and potency towards ER+ breast cancer cells than MCF10A depicting lower cell growth inhibitory effects on normal cells under B1SP treatment.

S7.

MCF7

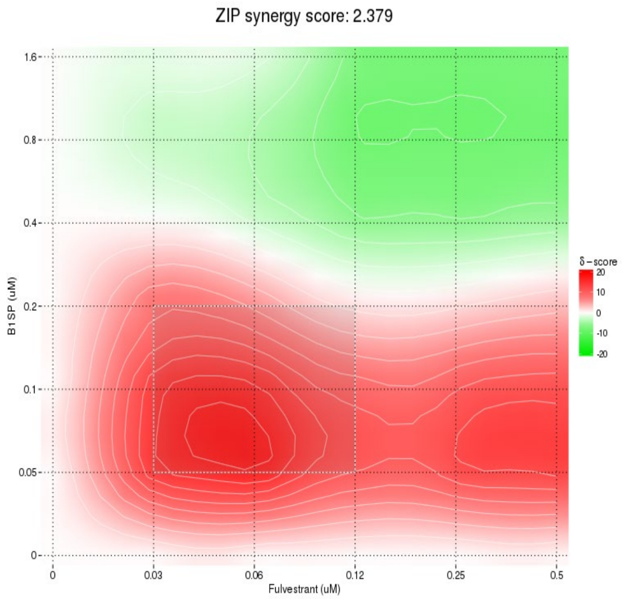

T47D

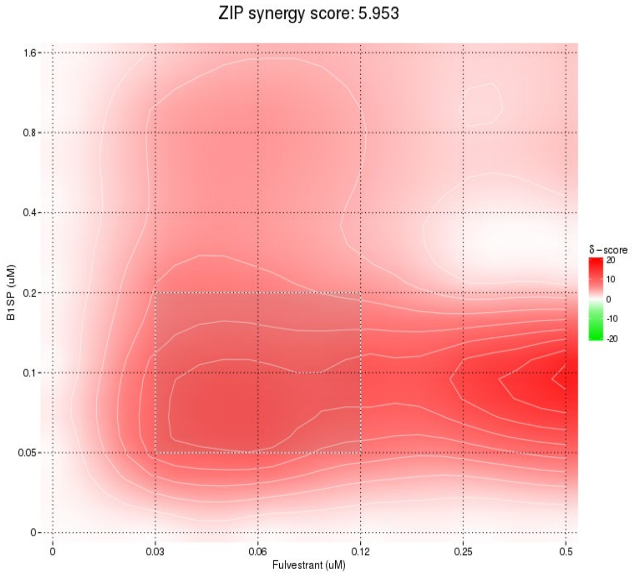

Figure S7. B1SP:Fulvestrant Synergy Map in MCF7 and T47D along with Z-scoring values developed with help of Synergy Finder online tool (red : Higher intensity depicts higher inhibition; green: Higher intensity depicts higher antagonistic effect.
